# The Impact of Caesarean Section on the Risk of Childhood Overweight and Obesity: New Evidence from a Contemporary Cohort Study

**DOI:** 10.1038/s41598-018-33482-z

**Published:** 2018-10-11

**Authors:** Gwinyai Masukume, Sinéad M. O’Neill, Philip N. Baker, Louise C. Kenny, Susan M. B. Morton, Ali S. Khashan

**Affiliations:** 1 The Irish Centre for Fetal and Neonatal Translational Research (INFANT), Department of Obstetrics and Gynaecology, University College Cork, Cork, Ireland; 2 College of Life Sciences, University of Leicester, Leicester, United Kingdom; 3 Department of Women’s and Children’s Health, Faculty of Health and Life Sciences, University of Liverpool, Liverpool, United Kingdom; 4 Centre for Longitudinal Research, University of Auckland, Auckland, New Zealand; 5 School of Public Health, University College Cork, Cork, Ireland

## Abstract

Caesarean section (CS) rates are increasing globally and exceed 50% in some countries. Childhood obesity has been linked to CS via lack of exposure to vaginal microflora although the literature is inconsistent. We investigated the association between CS birth and the risk of childhood obesity using the nationally representative Growing-Up-in-Ireland (GUI) cohort. The GUI study recruited randomly 11134 infants. The exposure was categorised into normal vaginal birth (VD) [reference], assisted VD, elective (planned) CS and emergency (unplanned) CS. The primary outcome measure was obesity defined according to the International Obesity Taskforce criteria. Statistical analysis included multinomial logistic regression with adjustment for potential confounders. Infants delivered by elective CS had an adjusted relative risk ratio (aRRR) = 1.32; [95% confidence interval (CI) 1.01–1.74] of being obese at age three years. This association was attenuated when macrosomic children were excluded (aRRR = 0.99; [95% CI 0.67–1.45]). Infants delivered by emergency CS had an increased risk of obesity aRRR = 1.56; [95% CI 1.20–2.03]; this association remained after excluding macrosomic children. We found insufficient evidence to support a causal relationship between elective CS and childhood obesity. An increased risk of obesity in children born by emergency CS, but not elective, suggests that there is no causal effect due to vaginal microflora.

## Introduction

Estimates from 121 countries reveal that Caesarean section (CS) rates increased from 6.7% in 1990 to 19.1% in 2014^1^. In 2015 the United States had a 32.0% CS rate^2^, Brazil 55.5%^3^ and England 26.5%^4^. Ireland experienced a similar rise in CS rates with an increase from 10.5%^1^ in 1990 to 31.4%^5^ in 2015.

There is no consensus regarding the optimal population-level CS rate, however, a systematic review suggested that rates up to 16% were associated with reduced maternal, neonatal and infant mortality^6^ and a further review reported reduction in mortality up to a 19% rate^7^. Multiple factors have driven the CS rate increase, including advanced maternal age at first childbirth, a decrease in vaginal births after Caesarean (VBAC)^8^, physician fear of litigation, maternal choice and access to private health insurance^9–13^.

Babies delivered by CS, particularly elective CS, are generally not exposed to their mother’s vaginal and faecal microbiota, which helps to shape the initial composition of an infant’s microbiota including that of the gut^14^. Infants born by elective CS have been found to have a gut microbiome that has low diversity and richness^15^. Some studies suggest that infants born by CS might have a gut microbiota that has a tendency to harvest more dietary nutrients, thereby predisposing them to being overweight or obese^16–18^.

There is epidemiologic evidence of an association between CS birth and subsequent excess body mass index (BMI) across the life course^19–22^. Although heterogeneity, confounding, publication bias and inability to account for elective versus emergency CS delivery were limitations in trying to unpack this association, a study using a sibling-control design found that those born by CS had significantly higher odds of being obese later in life compared to their siblings born vaginally^23^. It was, however, not possible in this sibling-control study to completely rule out confounding by the indications for CS, although the observed association was unlikely to be due to familial or genetic confounding^24^.

Childhood obesity and overweight are at epidemic levels globally^25^. Although the aetiology of childhood excess adiposity is multifactorial, given its serious complications^26^, the aim of this study was to investigate the relationship between obstetric mode of delivery and childhood overweight and obesity. We hypothesised that infants born by elective CS, because of the aforementioned reduced exposure to their mother’s vaginal and faecal flora would be at higher risk of being overweight or obese. In the most recent (2018) systematic review and meta-analysis considering the association between CS birth and childhood obesity (six cohorts), distinction between elective and emergency CS was not made^27^. In addition, small sample sizes have previously limited the evaluation of elective CS^28,29^. We aimed to investigate the potential confounding effect of macrosomia and/or large for gestational age (LGA) on the association between CS delivery and obesity. To our knowledge one previous study investigated this confounding effect^30^.

## Methods

### Data source and population sampled

The Growing Up in Ireland (GUI) study is a nationally representative infant longitudinal cohort (http://www.esri.ie/growing-up-in-ireland/), which recruited randomly 11134 infants born in Ireland from 1st December 2007 to 30th June 2008^31–33^. (Infants born during the months of July to November, inclusive, were not part of the GUI cohort.).

These children and their families had a baseline face-to-face questionnaire-based interview conducted by trained interviewers in participating households when the infants were approximately nine months old. Mother-infant pairs were subsequently followed-up by home interview when infants were three and five years old; follow-up continues. The response rates were as follows relative to most recent contact: at baseline interview (nine months) 64%, second interview (at three years) 91%, and at the third interview (at five years) 87%^31–33^. Children lost to follow-up tended to have unmarried mothers or mothers with lower educational attainment. In this study, children whose primary caregivers were not their mothers (*n *= 40, 0.36%) were excluded because the availability of potentially confounding variables such as age, maternal weight gain during pregnancy and health status predominantly pertained to mothers. In addition, children born by vaginal breech delivery (*n* = 41, 0.37%) and whose mode of delivery was unknown (*n* = 4, 0.04%) were also excluded, leaving 11,049 (99.2%) mother-infant pairs at baseline. Children born by vaginal breech delivery were excluded as they may differ from those born by vaginal cephalic delivery in important ways, for instance, they have a higher neonatal mortality rate^34^, moreover, we did not have enough numbers to include them as a separate category. Further details regarding the GUI study have been reported previously^31–33^.

### Exposure and outcome ascertainment

The primary exposure variable was obtained from mothers during the initial face-to-face interview when infants were nine months old by asking them, “What was the final mode of delivery?”, which has been demonstrated to be a robust method^35^. The delivery mode was grouped into four categories, namely normal vaginal delivery (VD), assisted VD and elective/planned and emergency/unplanned CS. Elective/planned and emergency/unplanned CS were mainly pre-labour or in labour respectively. The onset of labour contractions is significant because offspring microbial colonisation generally begins afterwards^36^. Children born by pre-labour CS would have had little to no exposure to vaginal microflora while children born by CS in labour were likely to have been exposed. Assisted VD constituted delivery by forceps or vacuum extraction. We used this classification system because it is well accepted clinically, and importantly, it allows us to test the main hypothesis that the association between CS and the increased risk of childhood obesity is due to differential exposure to vaginal microflora by mode of birth. The GUI study did not collect data on individual CS indications. Although the main focus of the present study is CS compared to normal VD, the assisted VD group is included in the analysis for completeness.

The child’s height and weight were measured by a trained interviewer using a validated standard measuring stick (Leicester portable height measure) and a medically approved weighing scale (SECA 835 digital weighing scales)^31–33^. BMI in kg/m^2^ was calculated for each child and each child was then classified as thin, normal, overweight or obese, according to the International Obesity Task Force (IOTF) - now World Obesity Policy & Prevention - system for boys and girls at age three and five years (please see Table 1 for the cut-offs for each category)^37,38^.

**Table 1:** International body mass index cut-off values by age and sex.

	3 years		5 years	
	Boys	Girls	Boys	Girls
Body mass index (kg/m^2^)				
Thin	<14.74	<14.47	<14.21	<13.94
Normal	≥14.74–<17.89	≥14.47–<17.56	≥14.21–<17.42	≥13.94–<17.15
Overweight	≥17.89–<19.57	≥17.56–<19.36	≥17.42–<19.30	≥17.15–<19.17
Obese	≥19.57	≥19.36	≥19.3	≥19.17

### Potential confounders

Data on the following potential confounders as reported in the literature^19–23^ were collected and included *a priori* in the analyses as presented in Table 2: maternal age, ethnicity, educational level, marital status, infant sex, birth weight, gestational age, parity, weight gain during pregnancy, preeclampsia and gestational diabetes. Parity defined as the total number of stillbirths and live births a woman has had was not available, however, we used the number of individuals currently in the study household who were a son/daughter of the mother as a proxy for parity. Birth weight centiles, adjusted for sex and gestational age, were calculated using the Bulk Centile Calculator for Ireland (please see Table 2 for the classification criteria into small, appropriate and large for gestational age; SGA, AGA and LGA respectively)^39^.

**Table 2:** Characteristics of the study population.

Characteristic	Overall n (%)	Normal vaginal delivery n (%)	Assisted vaginal delivery^a^ n (%)	Elective Caesarean section n (%)	Emergency Caesarean section n (%)
N	11049 (100)	6579 (59.5)	1596 (14.4)	1402 (12.7)	1472 (13.3)
**Maternal**					
Age, (years) median IQR	32 (28–36)	32 (28–35)	31 (27–35)	35 (31–37)	32 (28–35)
Ethnicity					
White	10266 (92.9)	6060 (92.1)	1530 (95.9)	1319 (94.1)	1357 (92.2)
Other	739 (6.7)	489 (7.4)	62 (3.9)	80 (5.7)	108 (7.3)
Missing	44 (0.4)	30 (0.5)	4 (0.3)	3 (0.2)	7 (0.5)
Marital status					
Married and living with husband	7421 (67.2)	4317 (65.6)	1007 (63.1)	1110 (79.2)	987 (67.1)
Married and separated from husband	210 (1.9)	131 (2.0)	27 (1.7)	24 (1.7)	28 (1.9)
Divorced/Widowed	134 (1.2)	78 (1.2)	16 (1.0)	20 (1.4)	20 (1.4)
Never married	3148 (28.5)	1955 (29.7)	534 (33.5)	235 (16.8)	424 (28.8)
Missing	136 (1.2)	98 (1.5)	12 (0.8)	13 (0.9)	13 (0.9)
Number of people in the household who are a son/daughter to the mother – ‘Parity’					
1	4508 (40.8)	2104 (32.0)	1208 (75.7)	325 (23.2)	871 (59.2)
2	3643 (33.0)	2424 (36.8)	274 (17.2)	583 (41.6)	362 (24.6)
3+	2898 (26.2)	2051 (31.2)	114 (7.1)	494 (35.2)	239 (16.2)
Missing	14 (0.1)	12 (0.2)	0 (0.0)	2 (0.1)	0 (0.0)
Gestational age, (weeks) mean (±SD)	39.5 (±2.1)	39.7 (±1.9)	40.1 (±1.6)	38.7 (±1.7)	38.9 (±3.0)
Missing	37 (0.3)	24 (0.4)	4 (0.3)	3 (0.2)	6 (0.4)
Weight gain during pregnancy, (kg) mean (±SD)	13.6 (±6.6)	13.4 (±6.6)	14.0 (±6.3)	13.8 (±6.4)	14.2 (±6.9)
Missing	1500 (13.6)	884 (13.4)	236 (14.8)	178 (12.7)	202 (13.7)
Pre-eclampsia	765 (6.9)	354 (5.4)	127 (8.0)	107 (7.6)	177 (12.0)
Gestational diabetes	316 (2.9)	151 (2.3)	42 (2.6)	61 (4.4)	62 (4.2)
**Offspring**					
Sex					
Male	5644 (51.1)	3253 (49.4)	885 (55.5)	702 (50.1)	804 (54.6)
Female	5405 (48.9)	3326 (50.6)	711 (44.5)	700 (49.9)	668 (45.5)
Birth weight, (g) mean (±SD)	3485 (±534)	3507 (±502)	3551 (±466)	3431 (±562)	3369 (±672)
Macrosomia (>4000 g)	1539 (13.9)	899 (13.7%)	228 (14.3%)	183 (13.1%)	229 (15.6%)
Missing	124 (1.1)	70 (1.1)	12 (0.8)	26 (1.9)	16 (1.1)
Birth weight centiles adjusted for sex and gestational age					
SGA < 10th centile	1552 (14.0)	910 (13.8)	236 (14.8)	175 (12.5)	231 (15.7)
AGA ≥ 10th centile ≤ 90th centile	8138 (73.7)	4932 (75.0)	1214 (76.1)	983 (70.1)	1009 (68.5)
LGA > 90^th^ centile	1199 (10.9)	643 (9.8)	130 (8.1)	215 (15.8)	211 (14.3)
Missing	160 (1.4)	94 (1.4)	16 (1.0)	29 (2.1)	21 (1.4)
Body mass index (kg/m^2^) at 3 years*					
Thin	445 (4.0)	275 (4.2)	56 (3.5)	48 (3.4)	66 (4.5)
Normal	6748 (61.1)	4037 (61.4)	1000 (62.7)	866 (61.8)	845 (57.4)
Overweight	1767 (16.0)	1038 (15.8)	249 (15.6)	227 (16.2)	253 (17.2)
Obese	506 (4.6)	280 (4.3)	67 (4.2)	73 (5.2)	86 (5.8)
Missing	1583 (14.3)	949 (14.4)	224 (14.0)	188 (13.4)	222 (15.1)
Body mass index (kg/m^2^) at 5 years*					
Thin	534 (4.8)	318 (4.8)	78 (4.9)	55 (3.9)	83 (5.6)
Normal	6459 (58.5)	3860 (58.7)	954 (59.8)	834 (59.5)	811 (55.1)
Overweight	1389 (12.6)	798 (12.1)	215 (13.5)	187 (13.3)	189 (12.8)
Obese	437 (4.0)	252 (3.8)	48 (3.0)	65 (4.6)	72 (4.9)
Missing	2230 (20.2)	1351 (20.5)	301 (18.9)	261 (18.6)	317 (21.5)

SD (Standard deviation), IQR (Interquartile range), SGA (Small for gestational age), AGA (Appropriate for gestational age), LGA (Large for gestational age).

^a^Vacuum or forceps.

*International Obesity Task Force age and sex-specific cut-offs.

Educational level not shown because of up to 14 overlapping categories that were challenging to recode into coherent mutually exclusive groups, missing data 10 (0.1%).

Breast feeding can be considered to be a mediator because mothers who gave birth by CS, particularly elective CS, are less likely to breastfeed^40^ and babies not breast fed are prone to future excess adiposity^41^. Variables such as the number of antibiotic courses during the last year, typical time to bed and the presence of a television in the child’s room have been associated with an increased risk of childhood obesity^42,43^. These variables including breast feeding were, however, not considered as confounders because they came after CS and cannot by definition confound the association between mode of birth and childhood obesity^44^.

### Missing data

Variables with missing data are as depicted in Table 2. The majority of key covariates had a low proportion of missing data. Importantly our outcome variable, BMI, had missing data either due to non-response or loss to follow-up which was equally distributed across the mode of delivery categories. Where a variable had a small amount of missing data (in this study all the key variables had <2% data missing) an extra category was added for example, ‘Ethnicity’ (1 = White; 2 = Other; 3 = Missing). It has been suggested that where missing data is minimal adding it as a missing category has a minimal impact on effect estimates^45^.

### Statistical analysis

Statistical analysis was conducted using Stata version 14SE (StataCorp LP College Station, TX). Frequency (n) and percent (%) were used to report categorical variables. The mean (standard deviation-SD) or median (interquartile range-IQR) were used to report numeric variables.

To evaluate the study hypothesis at ages three and five, we used multinomial logistic regression to calculate the adjusted relative risk ratio (aRRR) with 95% confidence intervals (CIs) with normal VD as the reference category and normal BMI as the base outcome. We also considered the association between mode of birth and transition of IOTF BMI category from three to five years (two time points); 0 = remained normal (base outcome), 1 = remained obese, 2 = became obese, 3 = became non-obese and 4 = other transition. For the multinomial regression models because the IOTF childhood BMI classification starts at two years of age^46^, we thus did not examine the association between mode of delivery and BMI at nine months age.

To explore if any associations could be explained by other factors we conducted sensitivity analyses by restricting analysis to SGA, AGA, LGA or non-macrosomic infants. Secondly we combined vaginal breech delivery with normal vaginal birth to form the reference category. We also performed subgroup analyses by infant sex, preterm birth (<37 weeks), restricting analysis to infants whose mothers did not have pre-eclampsia and to mothers <35 years old. Statistical significance was defined as a p-value < 0.05.

### Ethics statement

The GUI study received independent ethics approval from a Research Ethics Committee convened by the Department of Health and Children. Written informed consent was obtained from parents or guardians. All methods were performed in accordance with the relevant guidelines and regulations.

## Results

### Descriptive statistics

Of the 11049 infants, 8175 (74.0%) were delivered vaginally; most of these deliveries were by normal VD (59.5%) and the remainder were by assisted VD (14.4%). The rest of the deliveries (26.0%) were by CS; elective CS (12.7%) and emergency CS (13.3%) respectively (Table 2). The cohort had 51.1% boys and 48.9% girls; approximately 55% of deliveries by assisted VD and emergency CS were of boys. Of women who gave birth by elective CS just over half, 50.4%, were 35 years and older.

At birth, 13.9% of children were macrosomic (>4000 g); 10.9% were large for gestational age (population centiles). At three years of age, there were 1767 (18.7%) overweight and 506 (5.3%) obese children. At age five, the respective numbers were 1389 (15.8%) and 437 (5.0%).

Most children (n = 5030, 57.0%) remained within a normal BMI category between age three and five years whilst 175 (2.0%) remained obese. Two hundred and fifty six (2.9%) children who were obese at age three became non-obese (overweight, normal or thin) at age five and 262 (3.0%) children who were not obese at age three became obese at age five. Of the mothers who delivered vaginally, 13.2% were obese and of those who delivered by CS 21.5% were obese.

### Mode of delivery and BMI at age three years

There was an association between elective CS (aRRR = 1.32; [95% CI 1.01–1.74]) and emergency CS (aRRR = 1.56; [95% CI 1.20–2.03]) and the risk of obesity at age three years compared to the reference group of children delivered by normal VD (Table 3). The risk of being overweight at age three years was associated with emergency CS (aRRR = 1.23; [95% CI 1.04–1.44]) but not elective CS (aRRR = 1.06; [95% CI 0.90–1.25]).

**Table 3:** Mode of delivery and body mass index at age three years.

BMI category (normal BMI – base outcome)	Cases n (%)	RRR (95% CI)	p-value	AdjRRR (95% CI)**	p-value
Thin					
Normal vaginal delivery	275 (2.9)	reference		reference	
Assisted vaginal delivery	56 (0.6)	0.82 (0.61–1.10)	0.194	0.77 (0.56–1.05)	0.098
Elective Caesarean	48 (0.5)	0.81 (0.59–1.11)	0.2	0.84 (0.61–1.16)	0.299
Emergency Caesarean	66 (0.7)	1.15 (0.87–1.52)	0.336	1.11 (0.84–1.48)	0.456
Overweight					
Normal vaginal delivery	1038 (11.0)	reference		reference	
Assisted vaginal delivery	249 (2.6)	0.97 (0.83–1.13)	0.684	1.02 (0.87–1.20)	0.787
Elective Caesarean	227 (2.4)	1.02 (0.87–1.20)	0.815	1.06 (0.90–1.25)	0.467
Emergency Caesarean	253 (2.7)	1.17 (1.00–1.36)	0.056	1.23 (1.04–1.44)	0.013
Obese					
Normal vaginal delivery	280 (3.0)	reference		reference	
Assisted vaginal delivery	67 (0.7)	0.97 (0.73–1.27)	0.806	1.05 (0.78–1.39)	0.764
Elective Caesarean	73 (0.8)	1.22 (0.93–1.59)	0.154	1.32 (1.01–1.74)***	0.045
Emergency Caesarean	86 (0.9)	1.47 (1.14–1.89)	0.003	1.56 (1.20–2.03)	0.001

N for adjusted model = 9466. Multinomial logistic regression. BMI – Body mass index, RRR (Relative Risk Ratio), CI (Confidence intervals), Adj (Adjusted).

**Adjusted for maternal age, education, ethnicity, marital status, region, infant sex, gestational age, pre-eclampsia, gestational diabetes, parity. ***1.45 (1.10–1.91) when birth weight added.

There was no statistically significant association between elective CS and the risk of obesity at age three among AGA infants, (aRRR = 1.15; [95% CI 0.81–1.64]) (Supplementary Table S1). The analysis of AGA infants who were not macrosomic suggested that there was no association between elective CS and child obesity at age three years, (aRRR = 0.99; [95% CI 0.67–1.45]) (Supplementary Table S2). Among LGA infants there was an association between elective CS and the risk of obesity at age three years (aRRR = 2.01; [95% CI 1.10–3.67]) (Supplementary Table S3). The median birth weight for these LGA infants was 4200 g and their median birth centile was 97.6. SGA infants also drove the overall association, albeit just falling short of reaching statistical significance, (aRRR = 2.73; [95% CI 0.99–7.51]) (Supplementary Table S4). The median birth weight for these SGA infants was 3000 g and their median birth centile was 7.6. The p-value for the interaction term between delivery mode and birth centile categories in relation to obesity at age three years was <0.001.

There was an association between emergency CS (aRRR = 1.77; [95% CI 1.26–2.47]) and obesity when restricting to AGA non-macrosomic children (Supplementary Table S2).

For the observed elective CS effect, there was no statistically significant differential effect by sex, however, girls tended in the direction of having a greater effect size (p-value for interaction term was 0.093). Combining vaginal breech delivery with normal vaginal birth to form the reference category did not alter the results overall (data not shown). Excluding children of pregnancies complicated by pre-eclampsia or preterm birth and children of mothers less than 35 years of age did not affect the results overall (Supplementary Table S5).

### Mode of delivery and BMI at age five years

At age five, the association between elective CS and obesity was of borderline significance (aRRR = 1.30; [95% CI 0.98–1.73]) (Table 4); this association was not changed materially when the analysis was restricted to AGA non-macrosomic infants (aRRR = 1.26; [95% CI 0.86–1.84] (Supplementary Table S6), thus an association cannot be completely ruled out. Furthermore, there was an association between emergency CS and the risk of obesity (aRRR = 1.46; [95% CI 1.10–1.93]) (Table 4). There were no other statistically significant associations between mode of delivery and the remaining BMI categories. Restricting the analysis to AGA non-macrosomic children did not alter the observed association between emergency CS and the risk of obesity (Supplementary Table S6).

**Table 4:** Mode of delivery and body mass index at age five years.

BMI category (normal BMI – base outcome)	Cases n (%)	RRR (95% CI)	p-value	AdjRRR (95% CI)**	p-value
Thin					
Normal vaginal delivery	318 (3.6)	reference		reference	
Assisted vaginal delivery	78 (0.9)	0.99 (0.77–1.28)	0.954	0.95 (0.73–1.24)	0.697
Elective Caesarean	55 (0.6)	0.80 (0.60–1.08)	0.14	0.78 (0.57–1.06)	0.115
Emergency Caesarean	83 (0.9)	1.24 (0.96–1.60)	0.093	1.18 (0.90–1.54)	0.238
Overweight					
Normal vaginal delivery	798 (9.0)	reference		reference	
Assisted vaginal delivery	215 (2.4)	1.09 (0.92–1.29)	0.31	1.15 (0.97–1.37)	0.114
Elective Caesarean	187 (2.1)	1.08 (0.91–1.29)	0.366	1.13 (0.94–1.35)	0.19
Emergency Caesarean	189 (2.1)	1.13 (0.95–1.34)	0.181	1.18 (0.99–1.42)	0.066
Obese					
Normal vaginal delivery	252 (2.9)	reference		reference	
Assisted vaginal delivery	48 (0.5)	0.77 (0.56–1.06)	0.107	0.84 (0.60–1.16)	0.279
Elective Caesarean	65 (0.7)	1.19 (0.90–1.58)	0.219	1.30 (0.98–1.73)	0.072
Emergency Caesarean	72 (0.8)	1.36 (1.04–1.79)	0.027	1.46 (1.10–1.93)	0.009

N for adjusted model = 8819. Multinomial logistic regression. BMI – Body mass index, RRR (Relative Risk Ratio), CI (Confidence intervals), Adj (Adjusted).

**Adjusted for maternal age, education, ethnicity, marital status, region, infant sex, gestational age, pre-eclampsia, gestational diabetes, parity.

### Mode of delivery and BMI transition between ages three and five years

There was no association between elective CS and any BMI category transition (Supplementary Table S7). Those born by emergency CS had an increased risk of remaining obese from the age of three to five years (aRRR = 1.74; 95% CI 1.14–2.69]). Infants born by emergency CS also had an increased statistical risk of becoming non-obese (aRRR = 1.74; [95% CI 1.21–2.49]). Finally, emergency CS infants had an increased risk of making any other BMI category transition (aRRR = 1.20; [95% CI 1.04–1.38]).

Adding maternal weight gain in pregnancy (13.6% missing data) did not alter the interpretation of our results materially at age three or five years and transition between these ages.

## Discussion

### Main Findings

We investigated the association between CS birth, particularly elective CS, and the risk of childhood obesity using a large, prospective, nationally representative, longitudinal cohort study. In the multinomial logistic regression analysis we found insufficient evidence to support a causal relationship between elective CS and childhood obesity. Indications for emergency CS likely explained the increased risk of obesity observed in infants delivered via this mode, but not elective CS, suggesting that there is no causal effect due to vaginal microflora.

### Strengths and limitations

Firstly, the GUI study is a large and nationally representative sample due to the application of sampling weights. The major strength was that our main outcome, BMI, based on height and weight was collected prospectively by trained personnel using validated techniques thus minimising measurement error. In addition, BMI was classified using widely accepted international criteria which allows comparison with other populations. We did not assume that once an individual is classified as obese, they remain so at a future time point. This allowed us in addition to evaluate if the mode of delivery was associated with transition into or out of obesity between time points. The availability of an ample suite of variables to adjust for confounding also strengthened our study. For example, we included gestational diabetes which was not included by several previous studies^20^.

A limitation was the unavailability of maternal pre-pregnancy BMI which has been highlighted to attenuate effect estimates when included in models^20^. However this limitation was partially ameliorated because we had access to maternal gestational weight gain, an important variable in its own right, which has been suggested to be significantly correlated with maternal pre-pregnancy BMI^47^. Recall bias remains a concern because some key variables were collected sometimes a year after pregnancy. Our main predictor, mode of delivery, relied on maternal recall nine months post-partum. We can be confident however that this is likely to be accurate in the vast majority of cases given that a similarly designed and conducted population-based study from the United Kingdom, the Millennium Cohort Study reported that 94% of mothers recalled their mode of delivery nine months post-partum when compared to their hospital records^35^. Another aspect worth mentioning is that infants born during the months of July to November, inclusive, were omitted from the GUI cohort. This is a constraint because month of birth can serve as a proxy for specific seasonal environmental circumstances that can significantly influence future health^48^.

The classification of CS into elective and emergency, although addressing a limitation of previous studies, did not allow sufficient granularity of issues like whether the CS was purely on maternal request; these may differ from other elective CSs, or if membranes had ruptured prior to surgery (exposure of the fetus to vaginal microbiota). All the women classified in the elective CS group had pre-labour CS. Although it is likely that women in the emergency CS group mostly had in labour CS, we cannot rule out the possibility that some of them had pre-labour CS. This is unlikely to have influenced the elective CS result, especially in terms of our hypothesis which is based on pre-labour CS. Improving CS classification is an ongoing worldwide effort that is only gaining traction during this century^49^. There was lack of statistical power for some analyses, like the overweight analysis, however the RRRs were similar to previously reported associations. Given the consistency of our results we thus think there is merit in them.

Our proxy measure for parity, the number of individuals in the study household who were a son/daughter of the mother, assumed for instance that the mother had no biologic children outside the household. Despite the assumptions we made, the average number of children a mother had in the GUI cohort, infants born circa 2008, was 1.97 which is close to the 2008 reported total fertility rate for Ireland of 2.06^50^. Thus the proxy parity variable was likely to be accurate in most cases and capture birth order sufficiently in the models.

### Interpretation

The CS rate in this cohort was 26.0%, and is consistent with published national estimates of 25.6%^9^. This corroborates the national representativeness of the GUI cohort and the likely external validity of our findings. The 13.9% prevalence of macrosomia (>4000 g) however, was almost twice the 7.6% prevalence for the United States, another high-income country, during a similar time period circa 2008^51^. This suggests a highly obesogenic Irish milieu with high baseline levels of excess adiposity from birth.

We found high rates of childhood obesity and overweight, for comparison global obesity rates for girls and boys in 1975 were less than 1%^52^. The slightly lower prevalence of obesity at age five (5.0%) than at age three (5.3%) was in keeping with the natural obesity prevalence decline observed from approximately age two to 14 years^53^.

Approximately 80 studies of various designs (cohort, case control, cross sectional) and several systematic reviews have investigated the association between CS and offspring obesity^20,21,54^. Most of these studies found a positive association, however evaluation of this association was limited by publication bias, potential for residual confounding and moderate heterogeneity^20^. Studies which accounted for maternal pre-pregnancy weight and adjusted their analyses for a greater number of potential confounders reported effect sizes closer to the null^20^.

As reported by the previous systematic reviews and meta-analyses, we also found a small effect size (odds ratio/RRR < 1.50) before accounting for macrosomia in the association between CS birth and subsequent overweight and obesity^20,22^. We too found a greater association between CS birth and being obese than with being overweight^22^.

Few studies have been able to differentiate between emergency and elective CS^20,22,23^. However our finding that elective/planned CS is a risk factor for obesity at three years has been found previously in an American prospective cohort from Boston followed up largely during this century^55^. Nevertheless this study did not explore the potential confounding effect of macrosomia. Inability to account for elective and emergency CS calls into question the findings and conclusions of a sibling-control study^23^ which suggested a causal link between CS birth and future obesity. Another study with a sibling-control design, albeit also limited by inability to distinguish between elective and emergency CS, did not find an association between CS birth and higher BMI z score at age five years^56^. Unfortunately, the GUI cohort did not have data that allows sibling-cohort analysis.

The association between CS and obesity generally dissipates with increasing age, which can be attributed to attrition, greater interference by external factors such as antibiotic therapy or because of the natural decline in obesity prevalence from two to 14 years^22,23,53^. A study with follow-up to age twenty found higher overweight and obesity rates as well as higher concentrations of total and low-density lipoprotein cholesterol, leptin and apolipoprotein B in those born by CS^29^. It however remained unsettled if these unfavourable rates and markers of cardiometabolic disease could be attributed to CS birth itself or to the underlying reasons that necessitated CS birth.

Most studies have adjusted for birth weight^22^, however, a Canadian population-based survey is to the best of our knowledge the only study to specifically consider macrosomia, defined in that study as >4080 g^30^. Although a non-modifiable risk factor, it is important to highlight that emergency CS was associated with being overweight and obese at three years and being obese at five years. In addition, infants delivered by emergency CS were more likely to ‘transition’ between ages three and five, namely: remain obese, become non-obese (normal, overweight or thin), or have any other transition between the IOTF BMI categories.

As mentioned in the introduction, infants born by CS may have a microbiota that is more capable of harvesting dietary nutrients^16–18^. With emergency CS, membranes are more likely to have ruptured with consequent exposure of the infant to vaginal microbiota resulting in reduced odds of future obesity compared with elective CS infants. However finding a greater effect size for obesity following emergency CS, as previously reported^55^, suggests other mechanisms may be at play with emergency CS namely confounding by indication. Indeed a recent study suggested that the main mechanism driving the microbiota’s structure and function in infancy is body site and not mode of delivery^57^. Like we mentioned the natural history and drivers of being overweight or obese differ significantly by age. Although there is literature on adults^21^, some of which supports our findings, we focused our discussion on childhood at ages comparable to those in our study.

## Conclusion

We did not find enough evidence to support a causal relationship between elective CS and childhood obesity. An increased risk of obesity in children born by emergency CS, but not elective, suggests that there is no causal effect due to vaginal microflora and the association is likely to be explained by the underlying indications of emergency CS.

## Electronic supplementary material

Supplementary information

## Data Availability

The data that support the findings of this study are available from the Irish Social Science Data Archive (ISSDA), www.ucd.ie/issda, but restrictions apply to the availability of these data, which were used under license for the current study, and so are not publicly available. Bona fide researchers can apply for the data from ISSDA.
