# Supplementary material for: The Impact of Caesarean Section on the Risk of Childhood Overweight and Obesity: New Evidence from a Contemporary Cohort Study
Source: Sci Rep. 2018 Oct 11;8:15113. doi: 10.1038/s41598-018-33482-z (PMC6181954; doi:10.1038/s41598-018-33482-z)
Supplement: Supplementary file 1 — Supplementary information [file 41598_2018_33482_MOESM1_ESM.pdf]

Supplementary Information

The Impact of Caesarean Section on the Risk of Childhood Overweight and Obesity: New Evidence from a Contemporary Cohort Study

Gwinyai Masukume[1], Sinéad M O'Neill[1], Philip N Baker[2], Louise C Kenny[3], Susan MB Morton[4], Ali S Khashan[1,5,*]

[1]The Irish Centre for Fetal and Neonatal Translational Research (INFANT), Department of Obstetrics and Gynaecology, University College Cork, Cork, Ireland

[2]College of Life Sciences, University of Leicester, Leicester, United Kingdom

[3]Department of Women's and Children's Health, Faculty of Health and Life Sciences, University of Liverpool, Liverpool, United Kingdom

[4]Centre for Longitudinal Research, University of Auckland, Auckland, New Zealand

[5]School of Public Health, University College Cork, Cork, Ireland

Corresponding author:

*Ali Khashan: a.khashan@ucc.ie

Supplementary Table S1. Mode of delivery and body mass index at age 3 years, Appropriate for Gestational Age (AGA).

BMI category (normal BMI – base outcome)	n	RRR (95% CI)	p-value	AdjRRR (95% CI)**	p-value
Thin					
Normal vaginal delivery	188	reference		reference	
Assisted vaginal delivery	32	0.71 (0.48-1.04)	0.050	0.62 (0.42-0.92)	0.018
Elective Caesarean	27	0.63 (0.41-0.96)	0.067	0.62 (0.40-0.94)	0.023
Emergency Caesarean	36	0.92 (0.65-1.33)	0.977	0.84 (0.58-1.21)	0.354
Overweight					
Normal vaginal delivery	776	reference		reference	
Assisted vaginal delivery	203	1.04 (0.87-1.24)	0.601	1.08 (0.90-1.30)	0.384
Elective Caesarean	151	0.99 (0.81-1.20)	0.408	1.00 (0.82-1.21)	0.962
Emergency Caesarean	170	1.17 (0.97-1.41)	0.178	1.21 (0.99-1.47)	0.057
Obese					
Normal vaginal delivery	205	reference		reference	
Assisted vaginal delivery	52	1.02 (0.74-1.39)	0.923	1.03 (0.74-1.44)	0.861
Elective Caesarean	42	1.15 (0.69-1.37)	0.862	1.15 (0.81-1.64)	0.426
Emergency Caesarean	61	1.66 (1.15-2.08)	0.004	1.67 (1.22-2.29)	0.001

N for adjusted model = 7001. Multinomial logistic regression. BMI – Body mass index, RRR (Relative Risk Ratio), CI (Confidence intervals), Adj (Adjusted).
**Adjusted for maternal age, education, ethnicity, marital status, region, infant sex, gestational age, pre-eclampsia, gestational diabetes, parity.

Supplementary Table S2. Mode of delivery and body mass index at age 3 years, AGA, restricted to non-macrosomic.

BMI category (normal BMI – base outcome)		AdjRRR (95% CI)**	p-value
Thin			
	Normal vaginal delivery	reference	
	Assisted vaginal delivery	0.65 (0.44-0.97)	0.036
	Elective Caesarean	0.62 (0.40-0.94)	0.025
	Emergency Caesarean	0.83 (0.57-1.22)	0.341
Overweight			
	Normal vaginal delivery	reference	
	Assisted vaginal delivery	1.07 (0.88-1.31)	0.473
	Elective Caesarean	1.00 (0.82-1.23)	0.991
	Emergency Caesarean	1.15 (0.93-1.42)	0.195
Obese			
	Normal vaginal delivery	reference	
	Assisted vaginal delivery	1.08 (0.76-1.53)	0.682
	Elective Caesarean	0.99 (0.67-1.45)***	0.953
	Emergency Caesarean	1.77 (1.26-2.47)	0.001

N for adjusted model = 6321. Multinomial logistic regression. BMI – Body mass index, RRR (Relative Risk Ratio), CI (Confidence intervals), Adj (Adjusted).
**Adjusted for maternal age, education, ethnicity, marital status, region, infant sex, gestational age, pre-eclampsia, gestational diabetes, parity. ***0.96 (0.66-1.41) when birth weight added.

Supplementary Table S3. Mode of delivery and body mass index at age 3 years, Large for Gestational Age.

BMI category (normal BMI – base outcome)	n	RRR (95% CI)	p-value	AdjRRR (95% CI)**	p-value
Thin					
Normal vaginal delivery	21	reference		reference	
Assisted vaginal delivery	3	0.74 (0.48-1.04)	0.050	0.95 (0.26-3.44)	0.933
Elective Caesarean	4	0.66 (0.41-0.96)	0.067	1.04 (0.35-3.15)	0.939
Emergency Caesarean	8	1.34 (0.65-1.33)	0.977	1.28 (0.49-3.30)	0.614
Overweight					
Normal vaginal delivery	140	reference		reference	
Assisted vaginal delivery	27	1.00 (0.87-1.24)	0.601	1.01 (0.62-1.66)	0.963
Elective Caesarean	51	1.26 (0.81-1.20)	0.408	1.35 (0.90-2.01)	0.143
Emergency Caesarean	50	1.26 (0.97-1.41)	0.178	1.51 (1.00-2.29)	0.049
Obese					
Normal vaginal delivery	41	reference		reference	
Assisted vaginal delivery	10	1.27 (0.74-1.39)	0.923	1.19 (0.54-2.62)	0.666
Elective Caesarean	23	1.94 (0.69-1.37)	0.862	2.01 (1.10-3.67)	0.022
Emergency Caesarean	16	1.37 (1.15-2.08)	0.004	1.60 (0.80-3.20)	0.180

N for adjusted model = 1028. Multinomial logistic regression. BMI – Body mass index, RRR (Relative Risk Ratio), CI (Confidence intervals), Adj (Adjusted).
**Adjusted for maternal age, education, ethnicity, marital status, region, infant sex, gestational age, pre-eclampsia, gestational diabetes.

Supplementary Table S4. Mode of delivery and body mass index at age 3 years, Small for Gestational Age.

BMI category (normal BMI – base outcome)	n	RRR (95% CI)	p-value	AdjRRR (95% CI)**	p-value
Thin					
Normal vaginal delivery	56	reference		reference	
Assisted vaginal delivery	21	1.37 (0.80-2.33)	0.250	1.40 (0.79-2.51)	0.251
Elective Caesarean	16	1.72 (0.95-3.12)	0.075	1.36 (0.70-2.64)	0.368
Emergency Caesarean	20	1.43 (0.83-2.46)	0.198	1.25 (0.70-2.26)	0.454
Overweight					
Normal vaginal delivery	109	reference		reference	
Assisted vaginal delivery	18	0.60 (0.35-1.02)	0.060	0.64 (0.37-1.10)	0.108
Elective Caesarean	23	1.27 (0.77-2.09)	0.348	1.51 (0.87-2.60)	0.140
Emergency Caesarean	31	1.14 (0.73-1.76)	0.565	1.31 (0.83-2.07)	0.238
Obese					
Normal vaginal delivery	24	reference		reference	
Assisted vaginal delivery	5	0.76 (0.28-2.02)	0.581	0.90 (0.34-2.43)	0.840
Elective Caesarean	6	1.50 (0.60-3.78)	0.384	2.73 (0.99-7.51)	0.053
Emergency Caesarean	5	0.83 (0.31-2.22)	0.716	1.17 (0.43-3.18)	0.755

N for adjusted model = 1301. Multinomial logistic regression. BMI – Body mass index, RRR (Relative Risk Ratio), CI (Confidence intervals), Adj (Adjusted).
**Adjusted for maternal age, education, ethnicity, marital status, region, infant sex, gestational age, pre-eclampsia, gestational diabetes, parity.

Supplementary Table S5. Sensitivity analyses by various variables.

BMI category (normal BMI – base outcome)	AdjRRR (95% CI)**	p-value
Obese		
Normal vaginal delivery	reference	
Elective Caesarean*	1.39 (0.25-7.80)	0.707
Elective Caesarean**	2.70 (0.76-9.52)	0.123
Elective Caesarean***	1.17 (0.74-1.86)	0.497
Elective Caesarean****	1.28 (0.85-1.91)	0.237
Elective Caesarean*****	1.38 (0.95-1.99)	0.091

*pre-term < 37 weeks (N=386), **pre-eclampsia (N=460), *** mothers < 35 years old (N= 4601), ****males (N= 4795), *****females (N= 4671)

Supplementary Table S6. Mode of delivery and body mass index at age 5 years, AGA, restricted to non-macrosomic.

BMI category (normal BMI – base outcome)	AdjRRR (95% CI)**	p-value
Thin		
Normal vaginal delivery	reference	
Assisted vaginal delivery	0.98 (0.71-1.35)	0.899
Elective Caesarean	0.60 (0.40-0.89)	0.012
Emergency Caesarean	1.07 (0.76-1.50)	0.693
Overweight		
Normal vaginal delivery	reference	
Assisted vaginal delivery	1.16 (0.94-1.44)	0.168
Elective Caesarean	1.04 (0.83-1.31)	0.729
Emergency Caesarean	1.26 (1.00-1.58)	0.048
Obese		
Normal vaginal delivery	reference	
Assisted vaginal delivery	0.90 (0.60-1.35)	0.610
Elective Caesarean	1.26 (0.86-1.84)	0.231
Emergency Caesarean	1.56 (1.06-2.29)	0.024

N for adjusted model = 5889. Multinomial logistic regression. BMI – Body mass index, RRR (Relative Risk Ratio), CI (Confidence intervals), Adj (Adjusted).
**Adjusted for maternal age, education, ethnicity, marital status, region, infant sex, gestational age, pre-eclampsia, gestational diabetes, parity.

Supplementary Table S7. Mode of delivery and BMI category transition between ages three and five.

Transition (remained normal – base outcome)	**Cases n (%)**	**RRR (95% CI)**	**p-value**	**AdjRRR (95% CI)****	**p-value**
Remained obese					
Normal vaginal delivery	97 (1.1)	reference		reference	
Assisted vaginal delivery	18 (0.2)	0.74 (0.44-1.22)	0.237	0.79 (0.47-1.34)	0.385
Elective Caesarean	28 (0.3)	1.32 (0.86-2.02)	0.209	1.51 (0.98-2.33)	0.063
Emergency Caesarean	32 (0.4)	1.63 (1.08-2.45)	0.019	1.74 (1.14-2.69)	0.011
Became obese					
Normal vaginal delivery	155 (1.8)	reference		reference	
Assisted vaginal delivery	30 (0.3)	0.77 (0.51-1.14)	0.193	0.86 (0.57-1.29)	0.452
Elective Caesarean	37 (0.4)	1.09 (0.75-1.57)	0.652	1.15 (0.79-1.67)	0.464
Emergency Caesarean	40 (0.5)	1.28 (0.89-1.83)	0.183	1.37 (0.95-1.97)	0.096
Became non obese					
Normal vaginal delivery	138 (1.6)	reference		reference	
Assisted vaginal delivery	40 (0.5)	1.15 (0.80-1.65)	0.450	1.32 (0.91-1.93)	0.147
Elective Caesarean	34 (0.4)	1.12 (0.76-1.65)	0.553	1.20 (0.81-1.78)	0.357
Emergency Caesarean	44 (0.5)	1.58 (1.11-2.24)	0.011	1.74 (1.21-2.49)	0.003
Other transition					
Normal vaginal delivery	1833 (20.8)	reference		reference	
Assisted vaginal delivery	449 (5.1)	0.97 (0.85-1.11)	0.659	1.00 (0.87-1.14)	0.946
Elective Caesarean	383 (4.3)	0.95 (0.83-1.09)	0.494	1.00 (0.86-1.15)	0.946
Emergency Caesarean	431 (4.9)	1.16 (1.01-1.33)	0.031	1.20 (1.04-1.38)	0.013

N for adjusted model = 8819. Multinomial logistic regression. BMI – Body mass index, RRR (Relative Risk Ratio), CI (Confidence intervals), Adj (Adjusted). *p-value<0.05.
** Adjusted for maternal age, education, ethnicity, marital status, region, infant sex, gestational age, pre-eclampsia, gestational diabetes, parity.
